# Thermomechanical Analysis of Isora Nanofibril Incorporated Polyethylene Nanocomposites

**DOI:** 10.3390/polym13020299

**Published:** 2021-01-19

**Authors:** Cintil Jose, Chin Han Chan, Tan Winie, Blessy Joseph, Abhimanyu Tharayil, Hanna J Maria, Tatiana Volova, Francesco Paolo La Mantia, Didier Rouxel, Marco Morreale, David Laroze, Lovely Mathew, Sabu Thomas

**Affiliations:** 1 Newman College, Thodupuzha, Kerala 685585, India; cintiljose@gmail.com (C.J.); lovely.mathew@gmail.com (L.M.); 2 Faculty of Applied Sciences, Universiti Teknologi MARA, Shah Alam 40450, Malaysia; cchan_25@yahoo.com.sg (C.H.C.); tanwinie@salam.uitm.edu.my (T.W.); 3 International and Inter University Centre for Nanoscience and Nanotechnology, Mahatma Gandhi University, Kottayam, Kerala 686560, India; blessyprince14@gmail.com; 4 School of Energy Materials, Mahatma Gandhi University, Kottayam, Kerala 686560, India; abhimanyutharayil@gmail.com; 5 School of Chemical Sciences, Mahatma Gandhi University, Kottayam, Kerala 686560, India; hannavidhu@gmail.com; 6 Institute of Biophysics of the Siberian Branch of the Russian Academy of Sciences, Siberian Federal University, 79 Svobodnyi Av., 660041 Krasnoyarsk, Russia; volova45@mail.ru; 7 Dipartimento di Ingegneria, Università di Palermo, Viale delle Scienze, 90128 Palermo, Italy; 8 Consorzio INSTM, 50121 Firenze, Italy; 9 Institut Jean Lamour, UMR 7198 CNRS-Université de Lorraine, F-54500 Vandoeuvre-lès-Nancy, France; didier.rouxel@univ-lorraine.fr; 10 Facoltà di Ingegneria, Università degli Studi di Enna “Kore”, Cittadella Universitaria, 94100 Enna, Italy; marco.morreale@unikore.it; 11 Instituto de Alta Investigación, Universidad de Tarapacá, Casilla 7D, Arica 1000000, Chile; dlarozen@uta.cl

**Keywords:** polymer-cellulose nanocomposites, crystallization, mechanical properties, Avrami model

## Abstract

The research on cellulose fiber-reinforced nanocomposites has increased by an unprecedented magnitude over the past few years due to its wide application range and low production cost. However, the incompatibility between cellulose and most thermoplastics has raised significant challenges in composite fabrication. This paper addresses the behavior of plasma-modified polyethylene (PE) reinforced with cellulose nanofibers extracted from isora plants (i.e., isora nanofibrils (INFs)). The crystallization kinetics of PE–INF composites were explained using the Avrami model. The effect of cellulose nanofillers on tuning the physiochemical properties of the nanocomposite was also explored in this work. The increase in mechanical properties was due to the uniform dispersion of fillers in the PE. The investigation on viscoelastic properties confirmed good filler–matrix interactions, facilitating the stress transfer.

## 1. Introduction

Substantial progress in natural polymers inspired the design of cellulose-based materials, having a wide range of applications and considerably low manufacturing and production costs. Cellulose nanofibers and microfibers are driving the development of eco-friendly polymer nanocomposites due to their unique properties and tailorability [1]. Previously, cellulose fibers have been used as load-bearing components in highly functional composite materials [2,3]. Cellulosic fibers offer many advantages, like easy availability, abundance, renewability, low cost, tailorable mechanical properties, reduced energy consumption with low cost, and good environmental sustainability [4,5,6,7]. The poor compatibility between nanocellulose and thermoplastics makes the compounding process difficult, resulting in inferior properties in the composite [8,9]. In order to improve fiber–matrix interactions, various physical and chemical surface modification strategies, such as steam explosion, plasma modification, and graft polymerization, have been studied [8,10]. The surface modification also enhances the dispersion of the nanofillers in the polymer system [11,12]. The uniform and well-dispersed nanofillers form effective nucleating agents and affect the crystallization of the composite [13]. Polyethylene (PE) is widely used in various applications and is indispensable in modern life. PE is a preferred matrix material for composites, owing to its low cost, easy availability, and good processability [14].

Pasquini et al. reported that chemical modification of the cellulose fillers resulted in an increase in the overall properties of the low-density PE (LDPE) matrix, even though much improvement in the mechanical properties was not observed [15]. A similar enhancement in properties was also obtained using hexamethyldisiloxane-modified cellulose while developing low-density PE composites [16]. Chartrand et al. investigated the use of ferulic acid for esterification, as well as methacryloyl chloride and oleoyl chloride for acylation to modify microcrystalline cellulose. The modified cellulose was further compounded with the LDPE matrix without a free radical initiator. The acylation process ensured good dispersion of the cellulose material and showed improved modulus values, confirming the reinforcing effect [17]. The influence of surface-modified nanocellulose on the crystallization behavior of polycaprolactone (PCL) nanocomposites was studied by Roy et al. The work underlined the importance of the surface area and dispersion of nanofillers in the improvement of crystallization kinetics [18]. Bacterial cellulose, modified using the esterification process and maleic anhydride-grafted polypropylene, could improve the interfacial compatibility of the isotactic polypropylene. The increased crystallization observed was attributed to the homogeneous and heterogeneous nucleation [19].

In the present work, plasma-modified PE containing different cellulose nanofibril concentrations were prepared by melt mixing and compression molding to obtain a polymer nanocomposite material. The properties of the composites were studied using various characterization techniques like scanning electron microscopy (SEM), optical microscopy (OM), and atomic force microscopy (AFM). The isothermal crystallization studies were done to evaluate the influence of the nanofibril concentration on the kinetic parametric criteria of the PE matrix. The polymer–filler interactions were also analyzed through viscoelastic studies.

## 2. Materials and Methods 

### 2.1. Materials

The plasma-modified PE employed in this study, having a density of 0.95 g/cm^3^ and a melt flow index (MFI) of 7.0 g/10 min, was obtained from the Czech Republic (LLDPE, Dowlex 2631UE, The Dow Chemical Company, Midland, MI, USA). The cellulose nanofibrils (isora nanofibrils (INFs)), extracted from the *Helicteres isora* plant, exhibited a width of 20 ± 10 nm and an aspect ratio of 15. The isolation process was already reported by our group [20].

### 2.2. Preparation of the Isora Nanofibril (INF)-Reinforced PE Composite

The PE composites reinforced with INF were prepared by a melt mixing method with different concentrations of INFs (0.5, 1, and 3 wt%). The compounding was done using a Haake mixer at 60 rpm and 130 °C for 12 min. The compression molding was done with the help of an SHP-30 model hydraulic press at a pressure of 16.75 MPa for 5 min at 130 °C.

### 2.3. Differential Scanning Calorimetry (DSC)

A differential scanning calorimeter (TA DSC Q 1000, Milford, MA, USA) was employed to determine the melting phenomenon of the neat PE and INF–PE composites in a nitrogen environment. The samples were weighed, and around 4–8 mg was used for the experiment. The heating temperature rate was set to 10 °C/min over a temperature range of 30–170 °C. The melting and crystallization temperatures were taken from the graph. At least four samples were tested, and the reproducibility of the derived results was very good (deviation ≤ 1.5%).

### 2.4. Scanning Electron Microscopy (SEM)

The gold sputter-coated nanocomposites were analyzed using a JEOL JSM-820 model scanning electron microscope (Tokyo, Japan). Prior to imaging, the samples were kept in an oven at 60 °C for 12 h.

### 2.5. Atomic Force Microscopy (AFM)

The AFM measurements were carried out using an NT-MDT Solver PRO AFM (Moscow, Russia). The samples were analyzed under tapping mode with a resonance frequency of around 240 KHz.

### 2.6. Optical Microscopy (OM)

An optical microscope (Nikon Eclipse ME 600, Tokyo, Japan) was used to investigate the morphology of the PE composites. The samples were first heated to 170 °C at the rate of 10 °C/min and then kept at that temperature for 3 min. The cooling temperature rate was set to 20 °C/min, and the samples were then cooled to 117 °C. It was held at this temperature for 10 min, and the micrographs were captured.

### 2.7. Mechanical Characterization

The mechanical analysis was conducted as per ASTM D 5083 using a universal testing machine (Shimadzu Model AG1, Kyoto, Japan). The load cell used was 50 kN, and the crosshead speed was 10 mm/min. The gauge length was 50 mm. Five identical samples of a rectangular size were tested for each composition (deviation ≤ 5%).

### 2.8. Dynamic Mechanical Analysis

The dynamic mechanical properties of the nanocomposites were investigated using a dynamic mechanical thermal analyzer (DMTA) (GABO EPLEXOR 500 N, Netzsch, Selb, Germany). The tensile testing mode was employed at a frequency of 1 Hz. The experiment was carried out between a temperature range of 40–120 °C at a heating rate of 3 °C/min.

### 2.9. Rheological Characterization

The measurements were carried out using a parallel plate rheometer (Anton Paar, Graz, Austria) in the linear viscoelastic region (LVR). Dynamic strain sweep (DSS) tests at a constant frequency were conducted to confirm the occurrence of the strain amplitude in the LVR region before conducting the main rheological analysis.

### 2.10. Contact Angle Measurement

An SEO (Ansan, Republic of Korea) Phoenix contact angle testing instrument was used to carry out wettability studies via a sessile drop technique using triple distilled water. The readings were taken rapidly within 45–60 s of the addition of the liquid drop. The reproducibility of the results was satisfactory (deviation ≤ 4.5%).

### 2.11. Fourier Transform Infrared Spectroscopy (FTIR)

Fourier transform infrared spectra were recorded using a Shimadzu IR-470 IR spectrophotometer (Kyoto, Japan). The FTIR spectrum of each sample was obtained in the range of 400–4000 cm^−1^. The experiments were carried out with a resolution of 2 cm^−1^ and a total of 15 scans for each sample.

## 3. Results and Discussion

### 3.1. Isothermal Crystallization Behavior

**Figure 1:**
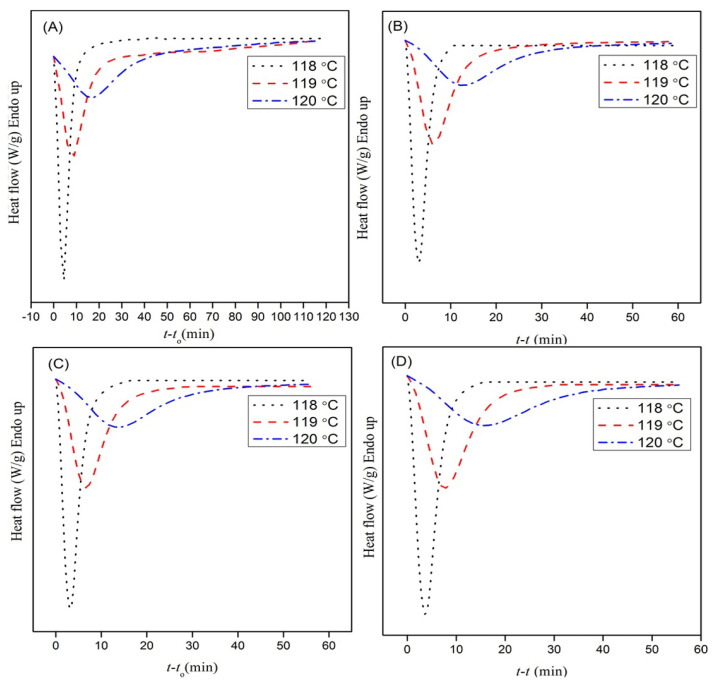
Dynamic sweep strain (DSC) thermograms of (**A**) neat polyethylene (PE), in addition to (**B**) 0.5 wt%, (**C**) 1 wt%, and (**D**) 3 wt% PE nanocomposites under different crystallization temperatures.

The crystallization studies of the nanocomposites were carried out under isothermal conditions. The crystallinity is an indication of how the addition of the filler into the PE matrix influences its crystallization behavior. Figure 1 shows the isothermal thermograms of the neat LDPE and INF–PE. The crystallization process occurred below the melting temperature T_m_, when the free energy of both the amorphous and crystalline phases were same. [21]. The driving force for nucleation at the surface of the nanoparticles was the free energy difference between the crystalline and amorphous phases [22]. With an increase in crystallization temperature, the exothermic peaks shifted to a greater value due to an increased nucleation density, as shown in Figure 1 [23,24]. All the exotherms shifted to shorter times with an increase in filler loadings at the same isothermal crystallization temperature. Thus, the nucleating effect of the INF filler material played a major role in improving the crystallization rate of the PE.

#### 3.1.1. Evaluation of Relative Crystallinity

**Figure 2:**
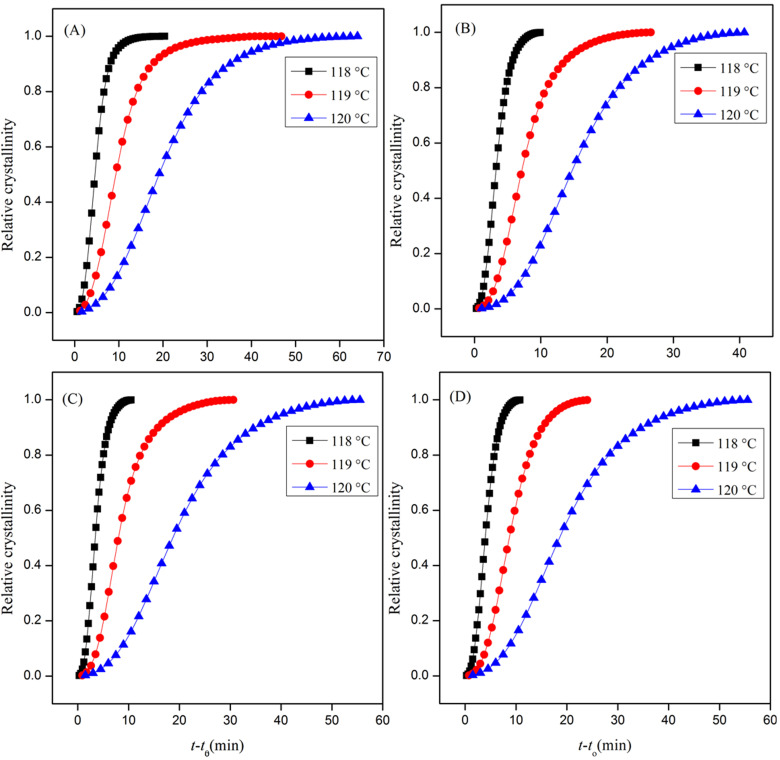
Relative crystallinity as a function of time for (**A**) pristine PE, in addition to (**B**) 0.5 wt%, (**C**) 1 wt%, and (**D**) 3 wt% PE nanocomposites at different crystallization temperatures.

The isothermal crystallization kinetics can be elucidated by evaluating the degree of crystalline conversion vs. time at a constant temperature. The relative crystallinity (*X*_t_) can be calculated using Equation (1); more specifically, *X*_t_ can be defined as follows [25]:

(1) $$X_{t}=\frac{\int_{T0}^{T}\frac{dHc}{dT}dT}{\int_{T0}^{Tw}\frac{dHc}{dT}dT}$$

where *T*_0_ and *T*_∞_ are the start and end of the crystallization temperatures and the heat flow rate is given by *dHc*/*dT*. At varied filler contents, the *X*_t_ versus time curves exhibited a characteristic sigmoidal shape. The S-shaped curve has an initial nonlinear part taken as the nucleation step of the crystallization process [26]. The primary crystallization corresponded to the linear part, and the secondary crystallization corresponded to the nonlinear part, which was due to the impregnation of the spherulites in the later stage of the crystal growth [27,28]. The isothermal crystallization behavior of the INF–PE composites was explained using the Avrami model. The relative crystallinity (*X*_t_) at different crystallization times could be calculated by partial integration of the crystallization exotherms, as shown in Figure 2.

#### 3.1.2. The Avrami Model

The change in relative crystallinity with time is better understood using the Avrami model [29,30,31]:

*X*_t_ = 1 − exp [−*K*^1/*n*^ (*t* − *t*_0_)]*^n^* (2)

where, *X*_t_ is the relative crystallinity at time *t*, *t*_0_ is the induction period, *K*^1/*n*^ represents the overall rate of crystallization (min^−1^), and *n* denotes the Avrami exponent that depends on the nucleation and geometry of the crystal growth.

Taking logarithms on both sides, Equation (2) can be transformed into

ln [−ln(1−*X*_t_)] = ln*K*^1/*n*^ + *n*ln (*t*−*t*_0_) (3)

**Figure 3:**
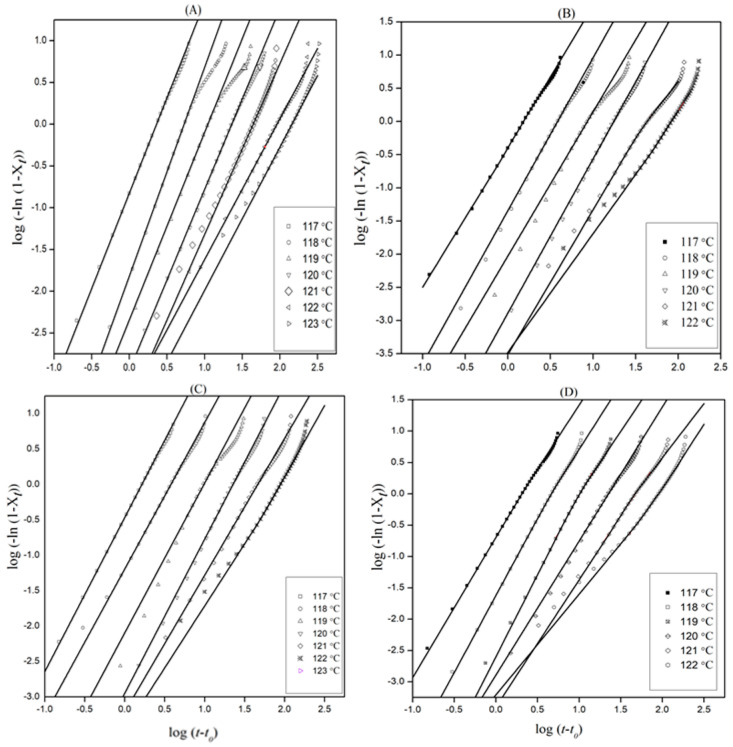
Avrami plots of (**A**) neat PE, in addition to (**B**) 0.5 wt%, (**C**) 1 wt%, and (**D**) 3 wt% PE nanocomposites under various crystallization temperatures.

The slope and intercepts of ln [−ln (1−*X*)] versus ln (*t*−*t*_0_) gives *n* and *K^1/n^*, respectively (Figure 3). The linear part of the isothermal thermograms that indicate 30–70% relative crystallinity gives the values of *n* and *K*.

The Avrami exponent *n*, the crystallization rate constant *K*, and the crystallization half-time *t*_0.5_ at different crystallization temperatures are listed in Table 1. Regression analysis was used to identify the confidence intervals of the overall rate constant and Avrami exponent. The curves obtained could be divided into the primary and the secondary crystallization processes. It was observed that the lines fitted for each section were nearly parallel, which confirmed that the nucleation mechanism and crystal growth geometries of different crystallization temperatures were similar for all the samples [32].

The common Avrami kinetics was followed by the initial stage of crystallization. When the spehrulites impinged during secondary crystallization, Avrami kinetics was not followed. The spherulites must be able to grow in free space for the Avrami model to be valid [33]. Thus, Avrami equations shed light on the type of nucleation and crystal growth. Three-dimensional crystal (spherical structure) growth resulting from an instantaneous athermal nucleation process occurred when *n* was close to three [33]. Truncated non-three-dimensional structures were observed when *n* was between two and three. The non-integral n value showed the combined effect of thermal and athermal nucleation [34].

As seen in Table 1, *n* ranged from 1.26 to 2.38 (*n* usually ranged between 2 and 2.38), indicating the evolution of 2D morphology to 3D spherical morphology, which is governed by both thermal and athermal nucleation [35]. The *n* values reported here were in close agreement with reports on the isothermal crystallization of PE [36,37]. The *n* values of the neat and PE nano composites were in the same range, indicating that the presence of the nanofiller did not induce any remarkable change in the isothermal crystallization mechanism.

The crystallization rate constant *K* decreased with an increase in *Tc*, indicating a decrease in the crystallization rate [38]. The addition of nanofillers enhanced the value of *K*, revealing the effectiveness of cellulose fibers as nucleating agents and thus promoting the crystallization of PE in the composite [35]. The half crystallization time (*t*_0.5_) listed in Table 1 showed a decreasing trend with the fiber content. It could be observed that the *t*_0.5_ values for the PE composite samples were lower than those for neat PE, suggesting that the incorporation of isora nanofillers facilitated the overall crystallization process. This indicates that INF restricted the mobility of the PE chains in the interspherulite regions [20,35]. Fillers can either enhance or retard crystallization, depending on the percolation concentration [39]. The linear portion of the curves (correlation coefficient *r*^2^ > 0.99) further suggests that the Avrami equation was valid for all samples [35].

#### 3.1.3. Activation Energy

The temperature dependence on the crystal growth rate, given by a Hoffman’s Arrhenius-like equation simplified in terms of the reciprocal half time [33], is

*t*_0.5_^−1^ α (−*E*_a_/*RT*_c_) (4)

where *E*_a_ represents the activation energy (J mol^−1^), *R* is the universal gas constant (JK^−1^ mol^−1^), and *T*_c_ is the crystallization temperature. The slope obtained from the semi-logarithmic plot of *t*_0.5_^−1^ versus (1/*Tc*) gives the activation energy (*E*_a_). The values are given in Table 2. There is reduction in the interfacial free energy, as reflected by a decrease in *E*_a_, thereby making the system crystallize easier and faster [35]. Table 2 shows the remarkably lower activation energy value of the PE composites compared with the neat PE. Thus, the nanofiller accelerated the crystallization process by acting as a nucleating agent [35,40,41]. The addition of nanofillers helped the PE chains to crystallize, which was evident from the reduction of the *E*_a_ value. A similar trend for PP/Si_3_N_4_ nanocomposites was reported by Hao et al. [23].

### 3.2. Morphological Analysis of the Nanocomposites

**Figure 4:**
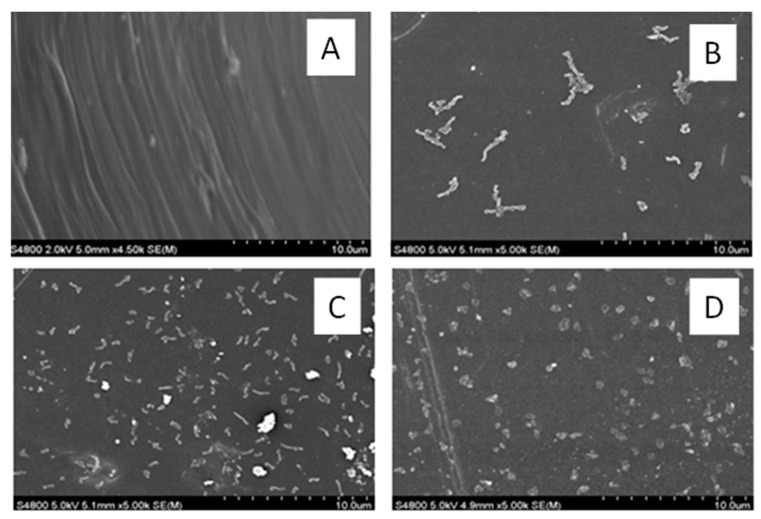
Surface morphology of the composites by scanning electron microscopy (SEM) analysis for (**A**) neat PE, in addition to (**B**) 0.5 wt%, (**C**) 1 wt%, and (**D**) 3 wt% INF-reinforced PE composites.

SEM analysis performed on the neat and composite systems showed good dispersion of the cellulose fibers in the polymer matrix (Figure 4).

**Figure 5:**
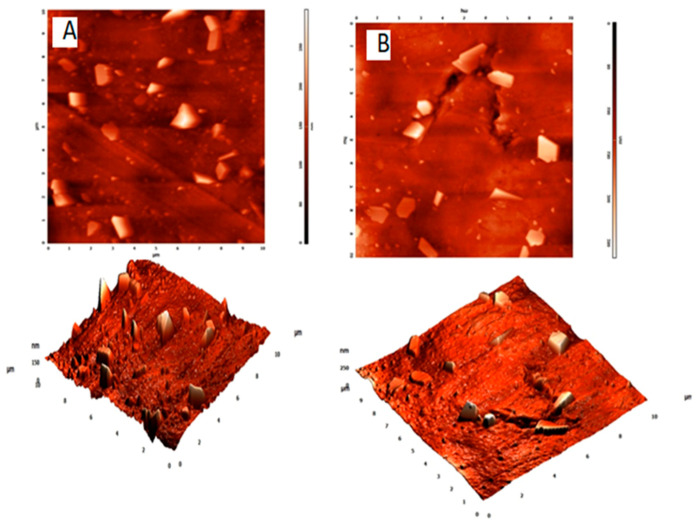
Atomic force microscopy images of (**A**) 1 and (**B**) 3 wt% INF-reinforced composites (phase image and image in 3D form).

The AFM images of the 1 and 3 wt% PE nanocomposites are shown in Figure 5. The nanofibrils were homogeneously dispersed, and no obvious signs of aggregation were seen.

**Figure 6:**
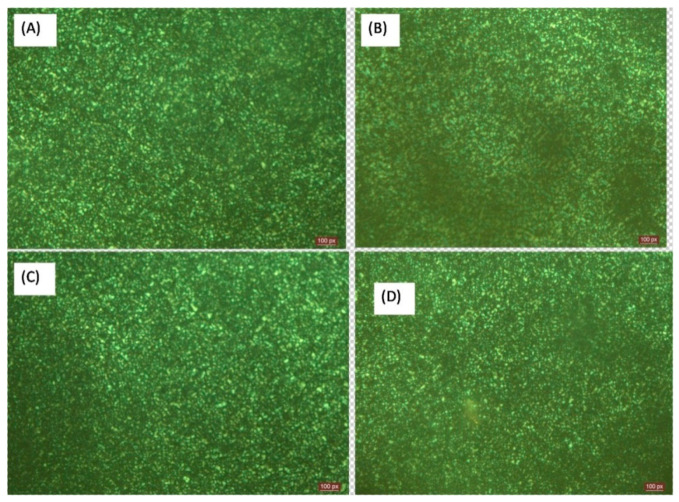
Optical micrographs showing spherulite growth for (**A**) neat PE, as well as the (**B**) 0.5 wt%, (**C**) 1 wt%, and (**D**) 3 wt% INF composites.

From the optical microscopy images (Figure 6), it can be inferred that the nucleation density and crystal size were not altered by the presence of nanofibrils [35].

### 3.3. Tensile Characterization

**Figure 7:**
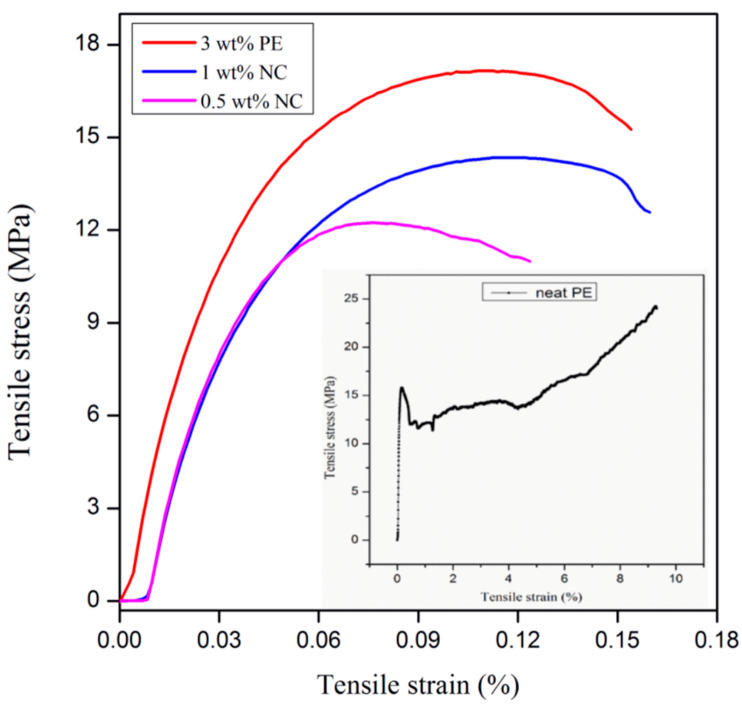
Stress–strain curve of INF–PE nanocomposites at different filler loadings.

From the stress–strain graph (Figure 7), it can be seen that with an increase in INF loading, the Young’s modulus increased, reaching a maximum at 1 wt%, and then dropped. The maximum tensile strength value was obtained for the 3 wt% INF. The increase in mechanical properties was due to the anchor effect, resulting from improved interaction between the filler and the matrix [20,35]. Similar behavior was observed for the polypropylene and PE composites containing cellulose [42]. Another reason for the improvement in mechanical properties could likely be the addition of nanofillers having high aspect ratios.

### 3.4. Rheological Characterization

#### 3.4.1. Viscoelastic Properties versus Strain

Viscoelasticity is a very important parameter that affects the processing of polymeric composite materials. It also helps to have an in depth look at the microstructure and the dispersion of the filler materials in the matrix directly in the melt state [35,43]. The filler–filler and polymer–filler interactions have a strong impact on the viscoelastic responses [44].

**Figure 8:**
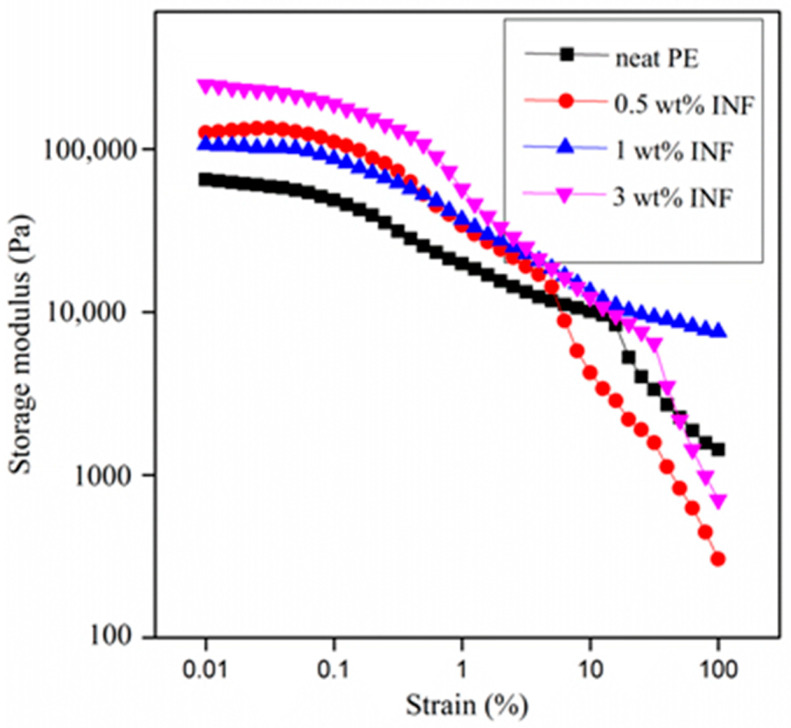
Storage modulus of PE nanocomposites at various filler loadings.

The storage modulus values for PE nanocomposites at various INF loadings is shown in Figure 8. The increase in the storage modulus (G’) with the INF concentration could be due to the increased contact at the polymer–filler interface, which enhanced stress transfer [14,20]. With an increase in strain, the bonds that are unstable tend to break, and only the stable bond contribution remains. This weakens more with the further increase in applied strain. The storage modulus of the system diminishes very quickly upon increasing the strain.

#### 3.4.2. Viscoelastic Properties versus Frequency

**Figure 9:**
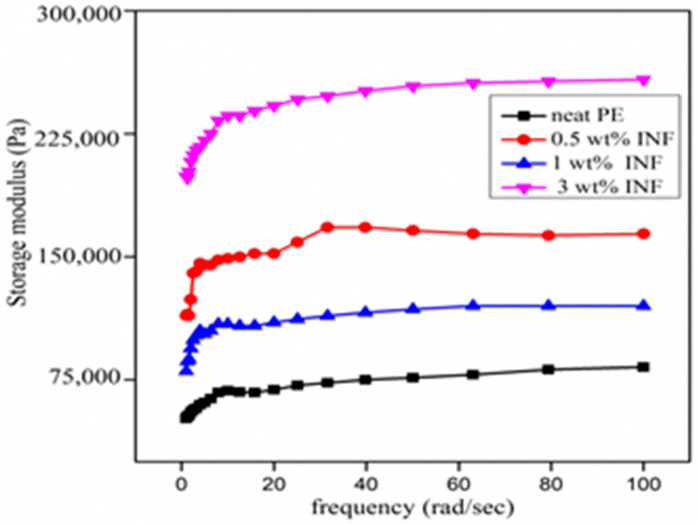
Frequency dependence of storage modulus for neat and INF-filled composites.

The frequency sweep measurements given in Figure 9 show an increase in moduli with the addition of nanocellulose. The moduli were independent of the frequency, indicating that the polymer systems did not relax within the time scales investigated. As in the strain sweep results, the G’ at 1% was lower than that at 0.5%, probably because of polymer–filler incompatibility. The strong interaction between the nanofiller and the polymer is evidenced by the excellent improvement in the storage modulus values [14]. The storage modulus of a nanocomposite tends to be independent of the frequency in the low frequency region, with elastic, solid-like characteristic behavior [35,45].

### 3.5. Dynamic Mechanical Analysis

**Figure 10:**
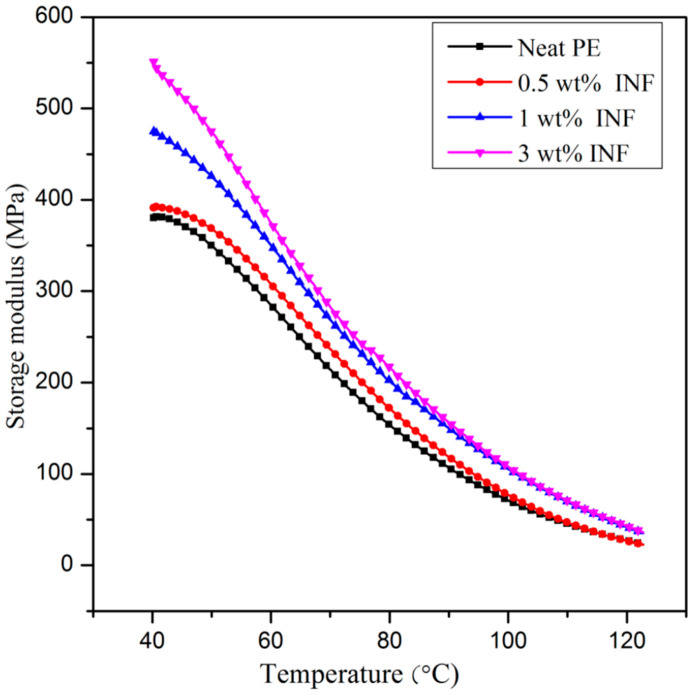
Storage modulus of INF-filled PE composites as a function of temperature.

The storage modulus vs. temperature graph is given in Figure 10. The storage modulus of the PE nanocomposites was higher than that of the neat PE. The stiffness of the PE matrix increased with the addition of the nanofiller, as a result of which the stress was better dissipated at the interface. As the temperature was increased, the storage moduli of all the samples showed a decreasing trend due to the progressive melting of the PE. The maximum E’ was obtained for 3 wt%. As the temperature increased, the ability of the molecular chains to move freely in the material enhanced, resulting in reduced storage moduli [46].

### 3.6. Contact Angle Measurements

The contact angle measurements give information about the actual wettability of a material. Parameters like Girifalco–Good’s interaction parameter, the spreading coefficient, work of adhesion, and interfacial free energy were quantified. With the addition of fillers, the hydrophilicity of the composite increased. With filler loading, the work of adhesion (WA) increased, due to the increased interfacial bonding and the presence of polar groups [35]. The nanocomposite material exhibited a decrease in the interfacial energy with increased INF loading. The spreading coefficient values shifted to lower negative values as the filler concentration increased. This was due to the increase in hydrophilic nature [35]. Girifalco–Good’s interaction parameter (Φ) decreased with the increase in filler loading. Table 3 summarizes the data from the contact angle studies.

### 3.7. FTIR Spectra

**Figure 11:**
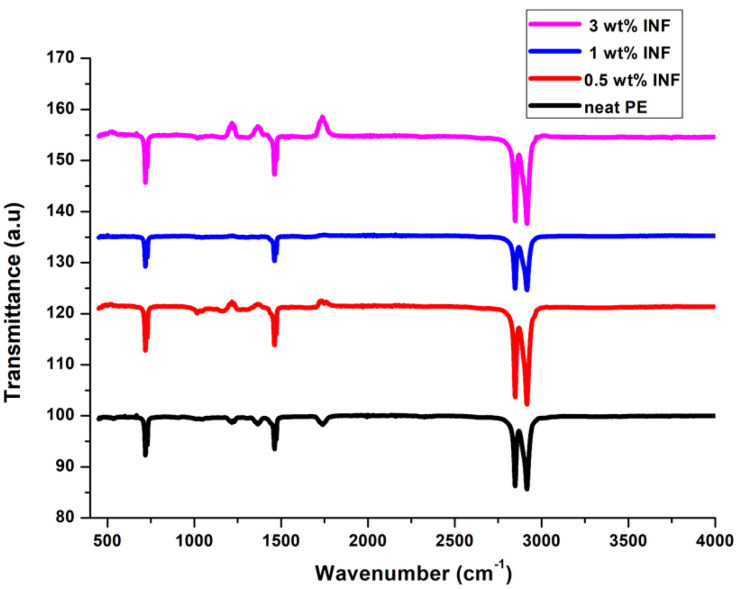
Infrared (IR) spectra of INF-filled PE composites as a function of temperature.

Figure 11 shows the FTIR spectra obtained for PE composites with different INF amounts. The peak near 3000 cm^−1^ relates to the C-H stretching vibrations of the PE chain in the composite. The peak at 1550 cm^−1^ was due to the OH bending of the absorbed water. As isora fibrils contain a large number of cellulosic hydroxyl groups, they can interact with the PE linkages to get a strong adhesion between the fiber and the matrix. The peak at 1600 cm^−1^ was due to the bending mode vibration of the absorbed water. It can be observed that the composites at different INF amounts basically showed the same absorption peaks, with marginal differences upon increasing the filler amount.

## 4. Conclusions

In the present study, the crystallization behavior of PE composites containing nanocellulose was explained by the Avrami model. The incorporation of nanofibrils significantly enhanced the crystallization kinetic constant, thus confirming the nucleating ability of isora nanofibrils. A gradual growth of two-dimensional morphology to a spherical three-dimensional morphology was observed for the spherulites. The *t*_0.5_ decreased with the increasing nanofiller content, as the crystallization proceeded faster. The values of *t*_0.5_ for the INF–PE composites were lower than those for the neat PE at a given crystallization time, signifying that the addition of nanofibrils could accelerate the overall crystallization process. As compared with the neat PE, the composite material had a lower *E*_a_ value. The influence of INF fibrils on the morphological, mechanical, viscoelastic, and wetting properties of PE were also studied. The morphology analysis confirmed that the dispersion of the fibers within the PE was adequate. Better interaction between the filler and the matrix was evidenced, as it led to the enhancement in properties. The entanglement between PE and isora nanofibrils, known as the anchor effect, was identified as the main contributor for the PE–INF fibril force. From the viscoelastic properties, it was found that the viscoelastic storage modulus of the composite material was higher than that of the neat PE, and this could be attributed to the reinforcement imparted by the filler that resulted in an increase in the stiffness, which led to a higher degree of interfacial stress transfer.

## Figures and Tables

**Table 1:** Avrami parameters of polyethylene isora nanofibril (PE–INF) nanocomposites.

Sample	*T*_c_ (°C)	*t*_1/2_ (min) Exptl	*t*_1/2_ (min) Calculated	Slope (*n*)	Δ*n*	*K*_A_^1/*n*^ (min)	Δ*K*_A_^1/*n*^ (min)	*r* ^2^
Neat	117	2.0	2.0	2.28	0.03	0.433	−0.0006	0.9997
	118	4..6	4.6	2.38	0.09	0.185	−0.0003	0.9983
	119	9.3	9.5	2.16	0.10	0.089	−0.0003	0.9968
	120	19.1	19.2	2.15	0.05	0.044	−0.0001	0.9990
	121	37.2	37.2	2.02	0.03	0.022	−0.0001	0.9997
	122	74	72.9	1.75	0.04	0.011	−0.0001	0.9991
	123	120	119.6	1.63	0.07	0.007	−0.0001	0.9974
0.5 wt% INF/PE	117	1.3	1.3	2.13	0.03	0.652	0.0004	0.9998
	118	3.2	3.3	2.25	0.07	0.261	−0.0002	0.9987
	119	7.1	7.3	2.17	0.11	0.116	−0.0005	0.9959
	120	14.7	14.8	2.23	0.07	0.057	−0.0048	0.9986
	121	35.2	35.7	1.98	0.05	0.023	−0.0002	0.9979
	122	69.1	69.1	1.96	0.04	0.007	−0.0001	0.9995
1 wt% INF/PE	117	1.4	1.4	2.17	0.02	0.611	0.0001	0.9999
	118	3.4	3.2	2.09	0.04	0.266	−0.0004	0.9995
	119	8.1	8.2	2.17	0.15	0.103	−0.0005	0.9932
	120	16.7	18.9	2.24	0.07	0.045	−0.0002	0.9986
	121	36.7	37.2	1.98	0.08	0.022	−0.0002	0.9977
	122	73.4	73.3	1.96	0.02	0.011	−0.0001	0.9999
	123	111.8	112.2	1.26	0.05	0.007	−0.0002	0.9963
3 wt% INF/PE	117	1.7	1.7	2.19	0.02	0.484	−0.0002	0.9999
	118	4.0	4.0	2.34	0.03	0.214	−0.0002	0.9996
	119	8.6	8.8	2.35	0.07	0.097	−0.0002	0.9984
	120	18.7	18.8	2.17	0.07	0.045	−0.0002	0.9978
	121	39.0	39.4	2.01	0.04	0.021	−0.0001	0.9992
	122	73.9	73.8	1.82	0.03	0.011	−0.0011	0.9994
	123	108	108.1	1.48	0.07	0.007	−0.0001	0.9954

**Table 2:** Activation energy of PE composites using Hoffman’s Arrhenius-like relationship.

Sample	Activation Energy (kJ mol^−1^)	*r* ^2^
Neat PE	−7.71	0.9899
0.5 wt% PE	−17.34	0.9968
1 wt% PE	−21.29	0.9873
3 wt% PE	−14.42	0.9848

**Table 3:** Wetting properties of INF–PE composites.

Sample	Contact Angle (Degrees)	Work of Adhesion (WA, mJ/m^2^)	Interfacial Energy γ_sl_ = γ_s_ + γ_l_ − WA (mJ/m^2^) *	Spreading Coefficient Sc = γ_s_ − γ_sl_ − γ_l_ (mJ/m^2^) *	Interaction Parameter Φ = [(1 + cosθ)γ_l_]/2(γ_s_ γ_l_)^1/2^ *
Neat PE	87.5	75.92	21.76	−69.68	1.63
0.5 wt%	81	83.07	19.78	−62.53	1.45
1 wt%	75	91.75	16.66	−53.85	1.29
3 wt%	68	100.04	15.22	−45.56	1.14

* The values were calculated based on the standard contact angle value obtained for the given sample.

## Data Availability

No new data were created or analyzed in this study. Data sharing is not applicable to this article.
